# Supplementary material for: MetaRibo-Seq measures translation in microbiomes
Source: Nat Commun. 2020 Jun 29;11:3268. doi: 10.1038/s41467-020-17081-z (PMC7324362; doi:10.1038/s41467-020-17081-z)
Supplement: Supplementary file 10 — Supplementary Data 7 [file 41467_2020_17081_MOESM10_ESM.zip › File2/Confidence_High_Taxonomy/84043_out.krona.html]

Javascript must be enabled to view this page.

members
magnitude
magnitudeUnassigned
count
unassigned
taxon
rank

84043\_out

3

2
3
superkingdom

phylum
3
976

200643
class
3

171549
3
order

171550
3
family

3
genus
239759

1288121
2
species

SRS012969\_contig\_number\_31127SRS023526\_contig\_number\_contig-100\_581.230234

328813
1
species

SRS077849\_contig\_number\_13388
